# Effects of test experience, closed-arm wall color, and illumination level on behavior and plasma corticosterone response in an elevated plus maze in male C57BL/6J mice: a challenge against conventional interpretation of the test

**DOI:** 10.1186/s13041-020-00721-2

**Published:** 2021-02-15

**Authors:** Hirotaka Shoji, Tsuyoshi Miyakawa

**Affiliations:** Division of Systems Medical Science, Institute for Comprehensive Medical Science, Fujita Health University, Toyoake, Aichi 470-1192 Japan

**Keywords:** Elevated plus maze, Open arm exploration, Anxiety-like behavior, Plasma corticosterone, C57BL/6J mice

## Abstract

The elevated plus maze test is a widely used test for assessing anxiety-like behavior and screening novel therapeutic agents in rodents. Previous studies have shown that a variety of internal factors and procedural variables can influence elevated plus maze behavior. Although some studies have suggested a link between behavior and plasma corticosterone levels, the relationships between them remain unclear. In this study, we investigated the effects of experience with a battery of behavioral tests, the wall color of the closed arms, and illumination level on the behavior and plasma corticosterone responses in the elevated plus maze in male C57BL/6J mice. Mice were either subjected to a series of behavioral tests, including assessments of general health and neurological function, a light/dark transition test, and an open field test, or left undisturbed until the start of the elevated plus maze test. The mice with and without test battery experience were allowed to freely explore the elevated plus maze. The other two independent groups of naïve mice were tested in mazes with closed arms with different wall colors (clear, transparent blue, white, and black) or different illumination levels (5, 100, and 800 lx). Immediately after the test, blood was collected to measure plasma corticosterone concentrations. Mice with test battery experience showed a lower percentage of open arm time and entries and, somewhat paradoxically, had lower plasma corticosterone levels than the mice with no test battery experience. Mice tested in the maze with closed arms with clear walls exhibited higher open arm exploration than mice tested in the maze with closed arms with black walls, while there were no significant differences in plasma corticosterone levels between the different wall color conditions. Illumination levels had no significant effects on any measure. Our results indicate that experience with other behavioral tests and different physical features of the maze affect elevated plus maze behaviors. Increased open arm time and entries are conventionally interpreted as decreased anxiety-like behavior, while other possible interpretations are considered: open arm exploration may reflect heightened anxiety and panic-like reaction to a novel situation under certain conditions. With the possibility of different interpretations, the present findings highlight the need to carefully consider the test conditions in designing experiments and drawing conclusions from the behavioral outcomes in the elevated plus maze test in C57BL/6J mice.

## Introduction

The elevated plus maze test has been widely used to measure anxiety-like behavior and evaluate potential therapeutic agents for anxiety-like traits in rats and mice [1–6]. This test is based on an approach/avoidance conflict resulting from the natural tendency of rodents to approach and avoid a potentially dangerous situation. Rodents normally avoid open arms in an elevated plus maze that are considered to be dangerous and instead tend to stay in closed arms for a longer time, and increased exploration of open arms is typically interpreted as a low level of anxiety. It is assumed that the behaviors can be induced by novelty, the presence of open spaces, and height in the testing environment [1, 6–8].

Elevated plus maze behavior can be influenced by a variety of internal factors and procedural variables, such as strain, sex, age, apparatus construction, and prior experience [8]. Some researchers have used a test/retest protocol with an intertest interval of at least one day and reported that prior experience with an elevated plus maze test reduces the exploration of open arms in the retest session [7, 9–15], whereas other reports indicate no changes in the exploration of open arms regardless of prior maze experience [1, 2, 16–18]. The decreased exploration of open arms during re-exposure to the maze is hypothesized to be involved in learning and memory of spatial information for open arms that are potentially dangerous [12, 13, 15, 19–21], locomotor habituation [10], and an altered receptor function [22, 23]. However, the effects of prior maze experience on subsequent behavior were not affected by manipulations of extramaze cues in DBA/2 mice [12], suggesting no importance of acquiring information about the test environment. In addition, previous reports showed that Swiss mice subjected to a holeboard test immediately before being tested in the elevated plus maze exhibited increased exploration of open arms [2], while repeated handling and exposure to an open field induced reduced exploratory behavior in the elevated plus maze in C57BL/6JOlaHs mice [24]. These findings suggest that such behavioral changes in the elevated plus maze may not necessarily require the learning and memory processes for previous experience in the same maze and can be induced by experience with different testing situations.

Wall transparency of closed arms is one of the factors for apparatus construction influencing elevated plus maze behavior. For example, it has been reported that higher open arm time and entries were observed in a maze with closed arms with transparent walls than with opaque walls in rats [25–27] and mice [28]. However, the effects of different color features of transparent walls or opaque walls on behavior have not been well studied. Illumination intensity in the testing environment has also been reported to be a significant factor that could affect open arm exploration. Rodents tested under higher illumination conditions showed reduced exploratory behavior in open arms or increased anxiety-like behavior compared with lower illumination conditions [23, 29–32], whereas some studies have shown that illumination levels did not affect the behavior [33, 34]. Thus, the effects of illumination on behavior remain inconclusive.

Corticosterone release resulting from hypothalamic–pituitary–adrenal (HPA) axis activation is necessary for homeostatic maintenance of the central and peripheral nervous systems to stressful or challenging situations [35]. Corticosterone levels have been considered to be associated with elevated plus maze behavior [1, 36–38]. In fact, the plasma corticosterone stress response in animals placed in the elevated plus maze is higher than that of animals left undisturbed in their home cages [36–38], and confinement to the open arms induces a greater increase in plasma corticosterone than confinement to the closed arms in rats [1]. These reports suggest that exposure to aversive stimuli from a novel situation, open space, and/or height causes a higher stress response. However, a previous study reported that plasma corticosterone levels showed no correlations with open arm exploration but significant correlations with risk-assessment behavior in Swiss-Webster mice that were allowed to freely explore an elevated plus maze [39]. Thus, little is still known about the relationships between plasma corticosterone levels and elevated plus maze behavior.

C57BL/6 mice are one of the most commonly used inbred mouse strains in behavioral and neuroscience research. Despite the extensive use of this strain, the influences of internal factors and procedural variables, e.g., experience with other behavioral tests and different physical features of the apparatus, on elevated plus maze behavior and plasma corticosterone response to the elevated plus maze have not been well studied in C57BL/6J mice. It is thus important to understand the influences of various experiences before the elevated plus maze test and apparatus conditions on elevated plus maze behavior and corticosterone response in this strain of mice. Additionally, it is interesting to explore whether corticosterone levels reflect “anxiety” levels that are naturally induced by exposure to potentially aversive situations in the elevated plus maze in C57BL/6J mice. In the present study, to examine the influences of experience with a battery of behavioral tests that did not include the elevated plus maze test on subsequent elevated plus maze behavior and plasma corticosterone responses in male C57BL/6J mice, animals were first subjected to a behavioral test battery, including an assessment of general health and neurological reflexes, a light/dark transition test, and an open field test, which have been extensively used to characterize the behavioral phenotypes of many strains of mutant and inbred mice in our laboratory [e.g., 40–43] and in other laboratories [e.g., 44–47]. Mice with and without experience with the behavioral test battery were tested in the elevated plus maze, and immediately after exposure to the maze, their blood was collected to measure plasma corticosterone levels. Additionally, to further explore the relationships between elevated plus maze behavior and plasma corticosterone levels after exposure to the elevated plus maze, the present study examined the influence of different test conditions, i.e., closed arm wall color and illumination level [25–34], on behavioral and endocrine responses.

## Methods

### Animals

Male C57BL/6J mice at 46–54 days old (n = 132 in total, from 4 different cohorts) were transported from Charles River Laboratories Japan, Inc. to our animal facility. The C57BL/6J strain has been maintained under The Jackson Laboratory's patented Genetic Stability Program at Charles River Laboratories Japan, Inc. After arrival at our facility, the mice were housed in groups (four per cage) in clear-transparent individually ventilated disposable cages (Innocage, 22.7 cm × 32.3 cm × 12.7 cm; Innovive, Inc., San Diego, USA) with paper bedding (PaperClean; Japan SLC, Inc., Shizuoka, Japan), which were placed in the middle or lower shelf of the rack in a ventilated rack system (Innorack; Innovive, Inc., San Diego, USA). The room was illuminated with fluorescent ceiling lights with a 12-h light/dark cycle (lights on at 7:00 AM). The illumination level in the cage in the middle shelf was approximately 25 lx. The cage position within the rack was kept through this study. The room temperature was maintained at 23 ± 2 °C. The mice were given access to food (CRF‐1; Oriental Yeast Co., Ltd., Tokyo, Japan) and water ad libitum throughout the study. The cages and paper bedding were changed every 10–14 days but not changed before and during the behavioral tests to avoid disturbing the mice. Animal care was carried out by a female laboratory technician. All experimental procedures were approved by the Institutional Animal Care and Use Committee of Fujita Health University.

The first cohort of mice (n = 32) was randomly assigned to either a test-battery-experience group or a no-test-experience group (n = 16 per group). Mice in the test-battery-experience group were subjected to a series of behavior tests that were performed in the following order: assessment of general health and neurological screening (2-day procedure; the day of start of the test battery was designated as test day 1; mice were 72–78 days old on test day 1 and 73–79 days old on test day 2), light/dark transition test (75–81 days old on test day 4), and open field test (77–83 days old on test day 6), with 2-day intertest intervals. The tests were performed in the same manner and the same order as previously described [e.g., 40–43; for details, see the “Behavioral test” section]. The no-test-experience group was left undisturbed until the start of the elevated plus maze test. Two to 4 days after the open field test (79–87 days old on test days 8–10), the mice of both groups were tested in the elevated plus maze, as described below. On each day of testing, the elevated plus maze test was performed in a manner counterbalanced for the groups to test approximately the same number of mice from each test condition (for details, see Additional File 1: Data file). Immediately after the elevated plus maze test, blood was collected from all the animals (for details, see the “Plasma corticosterone measurement” section).

The second cohort of naïve mice (n = 48) was tested at 73–82 days old in the elevated plus maze with closed arms of different colors (n = 12 per closed-arm color test condition): clear, transparent blue (the same color as in the experiment for the first cohort), white, and black (the first two were transparent and the last two were opaque; see Additional File 2: Fig. 1a and b). The test was conducted in the same manner as the experiment for the first cohort for 4 days. On each day, the test was performed in a manner counterbalanced for the test condition to test the same number of mice from each condition (for details, see Additional File 1: Data file), and then blood was collected immediately after the elevated plus maze test.

The third cohort of naïve mice (n = 36) was subjected to the elevated plus maze test under three different illumination levels: 5, 100, and 800 lx on the central platform in the maze (n = 12 per test condition, 72–80 days old). In this test, the same transparent blue closed arms as those in the experiment with the first cohort were used. For 3 days, the test was performed in a manner counterbalanced for the test condition to test the same number of mice from each condition on each day (for details, see Additional File 1: Data file). Immediately after the elevated plus maze test, blood was collected.

The fourth cohort of mice (n = 16) was used to measure basal levels of plasma corticosterone. The mice were either subjected to a behavioral test battery that did not include the elevated plus maze test in the same manner as in the first cohort of mice (n = 8) or were experimentally naïve (n = 8). The mice were 61–67 days old at the start of the test battery. Two days after the open field test, cages containing the mice with or without test battery experience (68–74 days old) were placed in the sound-attenuating room in which the elevated plus maze apparatus was set. Blood was collected from all the mice in a day in the same manner as the other cohorts of mice.

The naïve mice in all the cohorts had not been handled and were left undisturbed, except for routine cage maintenance conducted by the female laboratory technician. A male experimenter performed all the experiments, with the exception of the general health and neurological screen on test day 1 and the light/dark transition test on day 4 in the first cohort of mice that were carried out by the female technician. Consequently, the male experimenter conducted all procedures for the elevated plus maze test followed by blood sampling in all mice. In the elevated plus maze test, each mouse was picked up by the tail and removed from the home cage by the experimenter immediately before the test. The test-battery-experience group of the first cohort of mice received tail handling several times when subjected to a series of behavioral tests, whereas the other animals had not been habituated to tail handling before the elevated plus test.

### Behavioral test

Cages containing the mice to be tested were moved from a housing room to the sound-attenuating room in the morning at least 30 min before starting the test. The cages were put into the top shelf of a rack (90 cm × 35 cm × 88 cm) on which a top plate made of matte black acrylic was placed to avoid direct lighting from fluorescent ceiling lights in the testing rooms (the illumination level in the cage was approximately 5 lx), with the exception of the third cohort of mice that was put in the rack outside the sound-attenuating room because their test was conducted at different illumination levels. In all the cohorts of mice, the elevated plus maze test was performed between 9:45 AM and 1:15 PM. In the first cohort, a series of behavioral tests that did not include the elevated plus maze test were performed between 9:00 AM and 2:30 PM. After each test, the floors and walls of the testing apparatuses were cleaned with hypochlorous acid water to eliminate any olfactory cues. The cages were returned to the housing room after each test.

### General health and neurological screen

General health and neurological screen were conducted for 2 days. On day 1, physical characteristics and neurological reflexes (rectal temperature, body weight, righting reflex, whisker twitch reflex, ear twitch reflex, and visual placing reflex—a forepaw extension when lowered toward a visible surface) were assessed. To measure neuromuscular strength, mice were placed on a wire mesh that was then inverted, and the latency to fall from the mesh was recorded with a 60‐s cutoff time. On day 2, the forelimb grip strength was measured by holding the mice by their tails and lifting them so that their forepaws could grasp the wire grid of a grip strength meter (O'Hara & Co., Tokyo, Japan). The mice were gently pulled backward by the tail until they released the grid. The peak force applied by the forelimbs was recorded in newtons (N). Each mouse was tested three times. After the grip strength test, mice were placed in the box, and the reflex response to key jangling, the auditory stimulus, was recorded.

### Light/dark transition test

The light/dark transition test was performed as previously described [48] on test day 4. Four apparatuses set up in one of the sound-attenuating rooms were used to test four mice in a cage simultaneously. The apparatus consisted of a cage (21 cm × 42 cm × 25 cm) divided into two sections of equal size by a partition with a door (O’Hara & Co.). One chamber consisted of white plastic walls and was brightly illuminated (390 lx) by LED lights attached above the ceiling of the chamber. The other chamber had black plastic walls and was dark (2 lx). Both chambers had a white plastic floor. Mice were placed into the dark chamber and were allowed to move freely between the two chambers for 10 min with the door open.

### Open field test

The open field test was performed in an open field apparatus with the VersaMax Animal Activity Monitoring System (40 cm × 40 cm × 30 cm; Accuscan Instruments, Columbus, OH, USA). Eight apparatuses that were placed in a sound-attenuating room were used to test four to eight mice at the same time. The center area was illuminated to 100 lx by LED lights attached above the ceiling of each apparatus. The center area was defined as a 25 cm × 25 cm area. Each mouse was placed in one corner of the open field. Their behaviors, including the total distance traveled (cm), vertical activity (rearing, measured by counting the number of photobeam interruptions), time spent in the center area (s), and stereotypic counts (beam-break counts for stereotyped behaviors), were automatically recorded using an activity monitoring system for the entire 120-min period after the mice were placed in the apparatus.

### Elevated plus maze test

The elevated plus maze test was performed as previously described [49], using one elevated plus maze apparatus that was placed in a sound-attenuating room. Eight to 12 mice per day were tested between 9:45 AM and 1:15 PM each day to avoid any possible influences of diurnal changes in plasma corticosterone levels. The apparatus consisted of two open arms (25 cm × 5 cm) and two closed arms of the same size with 15-cm-high walls and a central square (5 cm × 5 cm) connecting the arms (O’Hara & Co.). The floor of the arms and central square were made of white PVC plates and were elevated to a height of 55 cm above the floor. To prevent mice from falling off the open arms, the arms were surrounded by a raised ledge (3 mm thick and 3 mm high, composed of transparent blue PVC). Arms of the same type were located opposite one another. The walls of the closed arms were made from one of the following: clear transparent walls, bluish transparent walls, matte white opaque walls, and matte black opaque walls, which were made of PVC plates (O’Hara & Co.). In the experiments with the first and second cohorts, the illumination level was set to 100 lx at the center of the maze with the closed arms with transparent blue walls (for details, see Additional File 6: Table 1). For the experiment with the third cohort of mice, which was tested at different illumination levels (5, 100, or 800 lx; four infrared LED lights attached to the ceiling were used to allow a video image-based analysis under the dim light condition). In each test, the cage was placed on the top plate of the rack, and the cage lid was removed. Each mouse was gently picked up by the tail and placed in an empty plastic open cage (18.2 cm × 25.0 cm × 13.9 cm). The housing cage was put back into the shelf, and the open cage containing a mouse was close to the central platform of the maze. Each mouse was then picked up by the tail and placed in the central square of the maze facing one of the closed arms. Immediately after placement, the experimenter pushed a start button of a remote controller of a video image analysis system attached to the wall of the sound-attenuating room and left the testing room. A camera was mounted on the ceiling above the central platform of the maze. The distance traveled (cm), number of total arm entries, time spent in the closed arms (s), time spent in the center area (s), percentage of open arm entries, and percentage of time spent in the open arms were automatically measured during a 10-min test period using the ImageEP program (ImageJ-based program developed by Tsuyoshi Miyakawa and the source code are freely available from the Mouse Phenotype Database website, http://www.mouse-phenotype.org/software.html, and from Git repository website, https://github.com/neuroinformatics [50]). Immediately following the end of the test, the experimenter returned to the room, put the mouse into the empty open cage, and then went to another sound-attenuating room adjacent to the testing room with the cage to take a blood sample. The floor and walls of the arms and central square were cleaned by hypochlorous acid water before each test to eliminate possible odors left by the different colored wall arms and the previous subject.

### Plasma corticosterone measurement

In the first, second, and third cohorts of mice, immediately after the elevated plus maze test (within approximately 1 min), blood was collected from the facial vein or submandibular vein using a Goldenrod Animal Lancet (MEDIpoint, Inc., NY, USA) into tubes containing 1 unit of sodium heparin (Wako Pure Chemical Industries Ltd., Osaka, Japan) and kept on ice. In the fourth cohort of mice, at least one hour after acclimation to the testing room where an elevated plus maze apparatus was set, blood was collected to measure basal levels of plasma corticosterone. The blood samples were centrifuged at 3000×*g* for 10 min at 4 °C. Supernatants were collected and stored at − 80 °C until measurement. Plasma corticosterone (CORT) concentrations were determined using a correlate-enzyme immunoassay kit (Assay Designs Inc., MI, USA) according to the manufacturer's protocol.

### Statistical analysis

Data were analyzed with Student’s *t*-test in the first and fourth cohorts of mice and one-way ANOVAs in the second and third cohorts of mice. The post hoc comparisons were performed by Student’s *t*-test with Bonferroni correction. Spearman’s rank correlation coefficients of the elevated plus maze behaviors with plasma corticosterone levels were calculated to examine relationships between the two measures. The significance level was set at 0.05. The statistical analysis was performed using SAS University Edition software (SAS Institute Inc., NC, USA).

## Results

**Fig. 1:**
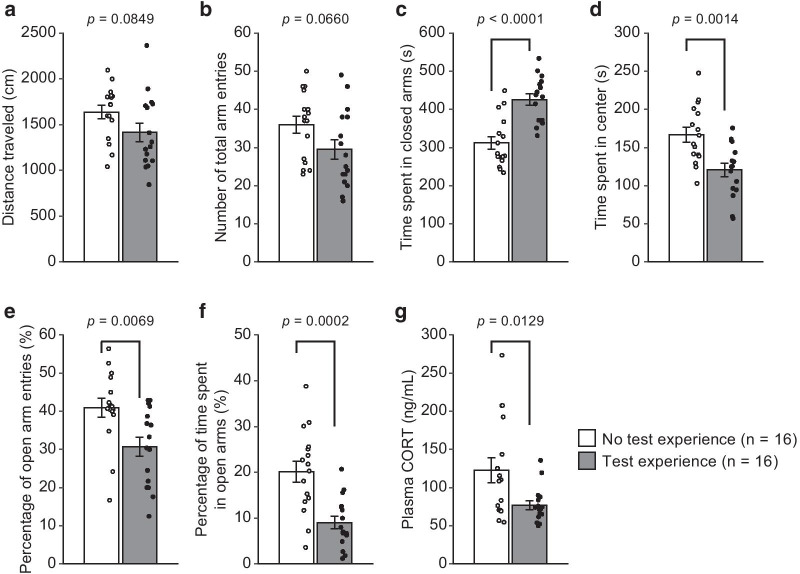
Effects of test experience on behaviors and plasma corticosterone levels in the elevated plus maze in male C57BL/6J mice. Mice that were subjected to behavioral tests other than the elevated plus maze test (n = 16) and mice with no test experience (n = 16) were tested in an elevated plus maze. **a** Distance traveled (cm), **b** number of total arm entries, **c** time spent in closed arms (s), **d** time spent in center area (s), **e** percentage of open arm entries (%), and **f** percentage of time spent in open arms (%) were measured. **g** Plasma corticosterone levels (ng/mL) of the mice exposed to the elevated plus maze were determined. Values are the means ± SEM

Mice with test battery experience showed slightly less distance traveled and fewer total arm entries (Fig. 1a: *t*_30_ = 1.78, *p* = 0.0849; Fig. 1b: *t*_30_ = 1.91, *p* = 0.0660, respectively), spent significantly more time in the closed arms (Fig. 1c: *t*_30_ = 5.06, *p* < 0.0001) but spent less time in the center area (Fig. 1d: *t*_30_ = 3.52, *p* = 0.0014), entered the open arms less frequently (Fig. 1e: *t*_30_ = 2.90, *p* = 0.0069), and spent less time in the open arms (Fig. 1f: *t*_30_ = 4.17, *p* = 0.0002) than mice with no prior experience. Plasma corticosterone concentrations after exposure to the elevated plus maze were lower in the test-battery-experience group of mice than in the group of mice with no prior experience (Fig. 1g: *t*_30_ = 2.64, *p* = 0.0129), while the basal plasma corticosterone levels in the mice with and without test battery experience did not differ from each other (Additional File 3: Fig. 2: *t*_14_ = 0.45, *p* = 0.6586). In this cohort of mice, the elevated plus maze test was conducted 2–4 days after the open field test, and therefore, whether the different intertest intervals contributed to behavioral differences between groups was examined. Two-way ANOVAs with group and test day as factors showed no significant group × test day interactions on any measures (Additional File 4: Fig. 3), which indicated that group differences in each behavior were not dependent on the test day.

**Fig. 2:**
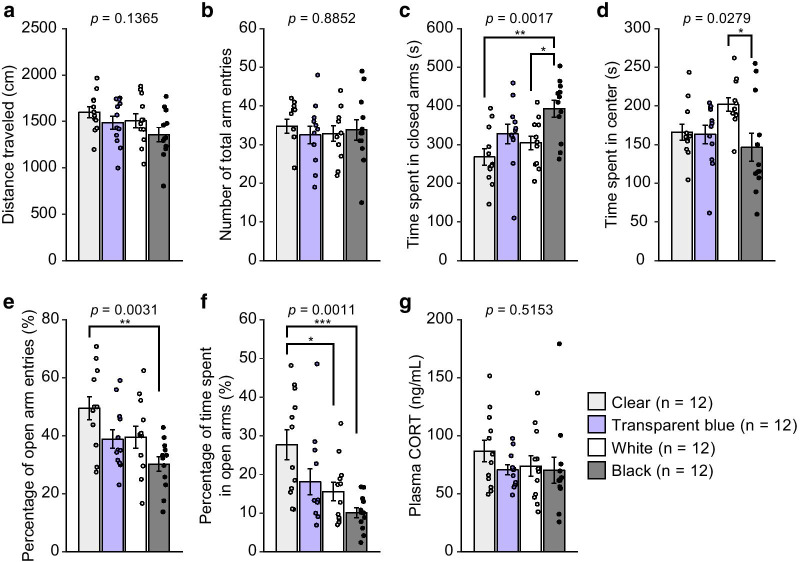
Effects of closed arm wall color on behaviors and plasma corticosterone levels in the elevated plus maze in male C57BL/6J mice. Mice were tested in an elevated plus maze with closed arms of any one of four colors (clear, transparent blue, white, and black; n = 12 per test condition). **a** Distance traveled (cm), **b** number of total arm entries, **c** time spent in closed arms (s), **d** time spent in center area (s), **e** percentage of open arm entries (%), **f** percentage of time spent in open arms (%), and **g** plasma corticosterone concentration (ng/mL) after the test were measured. Values are the means ± SEM. When ANOVA indicated a significant effect (p < 0.05), group comparisons were made using Student's t-test: *p < 0.05, **p < 0.01, ***p < 0.001 after Bonferroni correction

For the second cohort of mice, there were significant effects of closed-arm wall color on several behavioral measures (Fig. 2a–f), including time spent in the closed arms (Fig. 2c: *F*_3,44_ = 5.92, *p* = 0.0017), time spent in the center area (Fig. 2d: *F*_3,44_ = 3.33, *p* = 0.0279), percentage of open arm entries (Fig. 2e: *F*_3,44_ = 5.37, *p* = 0.0031), and percentage of time spent in the open arms (Fig. 2f: *F*_3,44_ = 6.39, *p* = 0.0011). No significant effects of closed-arm wall color were found on distance traveled (Fig. 2a: *F*_3,44_ = 1.94, *p* = 0.1365), number of total arm entries (Fig. 2b: *F*_3,44_ = 0.22, *p* = 0.8852), or plasma corticosterone levels (Fig. 2g: *F*_3,44_ = 0.77, *p* = 0.5153). Post hoc comparisons showed that mice subjected to the black wall condition spent more time in the closed arms and exhibited lower percentages of open arm entries and open arm time than mice in the clear transparent condition (*p* = 0.0002, *p* = 0.0002, and *p* < 0.0001, respectively). Mice in the black wall condition also spent more time in the closed arms than mice in the white and bluish transparent wall conditions, although the difference between the black and bluish transparent wall conditions did not reach significance after Bonferroni correction (*p* = 0.0058 and *p* = 0.0383, respectively). Mice in the black wall condition spent less time in the center area than mice in the white wall condition (*p* = 0.0037). Mice in the clear transparent wall condition tended to enter the open arms more frequently and spend more time in the open arms than mice in the bluish transparent and white wall conditions (for percentage of open arm entries, clear vs. bluish transparent and white, *p* = 0.0323 and *p* = 0.0431; for percentage of open arm time, clear vs. bluish transparent and white, *p* = 0.0251 and *p* = 0.0052), although almost all the statistical results did not reach significance after Bonferroni correction.

**Fig. 3:**
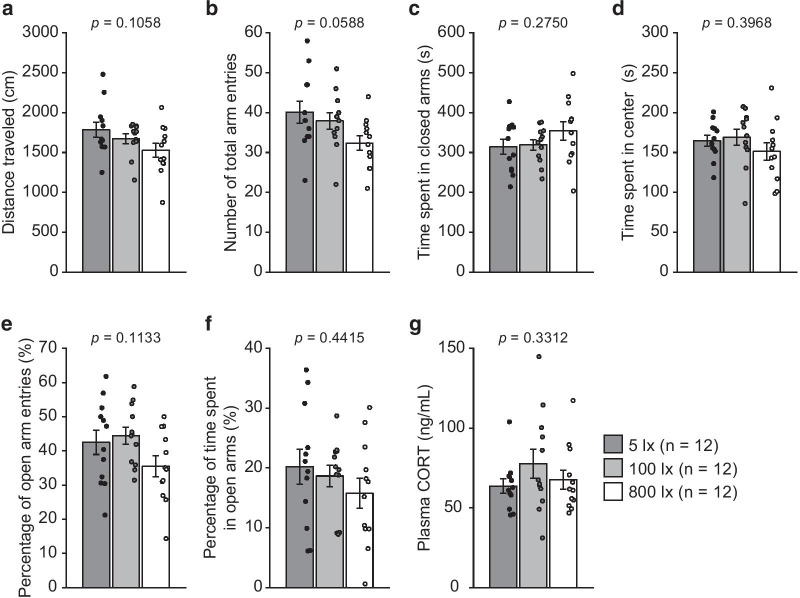
Effects of illumination level on behaviors and plasma corticosterone levels in the elevated plus maze in male C57BL/6J mice. Mice were tested in the elevated plus maze under any one of three lighting conditions (illumination levels of 5, 100, and 800 lx, on the center area of the maze). **a** Distance traveled (cm), **b** number of total arm entries, **c** time spent in closed arms (s), **d** time spent in center area (s), **e** percentage of open arm entries (%), **f** percentage of time spent in open arms (%), and **g** plasma corticosterone concentration (ng/mL) after the test were measured. Values are the means ± SEM

In the third cohort of mice, there were no significant effects of illumination level on any behavioral measures or plasma CORT level (Fig. 3a–g: distance traveled, *F*_2,33_ = 2.41, *p* = 0.1058; number of total arm entries, *F*_2,33_ = 3.09, *p* = 0.0588; time spent in closed arms, *F*_2,33_ = 1.34, *p* = 0.2750; time spent in center area, *F*_2,33_ = 0.95, *p* = 0.3968; percentage of open arm entries, *F*_2,33_ = 2.33, *p* = 0.1133; percentage of time spent in open arms, *F*_2,33_ = 0.84, *p* = 0.4415; plasma CORT level, *F*_2,33_ = 1.14, *p* = 0.3312).

To examine the relationships between the elevated plus maze behaviors and plasma corticosterone levels, Spearman’s rank correlation coefficients of each behavioral measure with plasma corticosterone levels were calculated using the data from the groups of mice tested under the different test conditions (for each group, n = 32–48; Additional File 5: Fig. 4a–r) and from all the mice (n = 116; Additional File 5: Fig. 4s–x). The correlation analyses showed that there was a significant negative correlation of plasma corticosterone level with time spent in the closed arms in the first cohort of mice with and without test experience (Additional File 5: Fig. 4c), while there were no any significant correlations between plasma corticosterone levels and behavioral measures in the group of all 116 mice (Additional File 5: Fig. 4s–x).

## Discussion

The present study investigated the influences of experience with a battery of behavioral tests that did not include the elevated plus maze test, closed-arm wall color, and illumination level on elevated plus maze behavior during a 10-min period from the elevated plus maze test and plasma corticosterone levels immediately after exposure to the maze in male C57BL/6J mice. The results showed that experience with some types of behavioral tests induced decreases in both exploration of the open arms of the maze and plasma corticosterone response, while testing in closed arms of different wall colors affected behavior but not plasma corticosterone levels, and illumination level had no significant effects on any measures. Additionally, correlation analyses showed a negative correlation of close arm time with corticosterone levels in the cohort of mice with and without test experience, whereas in a group consisting of all mice used in this study, there were no significant correlations between them. These findings indicate that experience with other behavioral tests and test apparatus construction are significant factors affecting elevated plus maze behaviors, but the relationships between the behaviors and plasma corticosterone levels may depend on the test situation and thus remain unclear in C57BL/6J mice.

The present results suggest that reduction in open arm exploration can occur due to prior exposure to test apparatuses other than the elevated plus maze, which seems to be consistent with findings of previous studies [2, 24]. Our present study and other previous studies [2, 24] may provide an additional or alternative explanation for the reduction in open arm exploration in mice with prior experience in previous studies using the test/retest protocol, in which the reduction of open arm exploration in a retest was considered to be based on learning of open arm avoidance during the first elevated plus maze test [12, 13, 15, 19–21]. The reduced open arm exploration in the retest may be due to exposure to the elevated plus maze apparatus itself and/or due to previous experience(s), other than reexposure to the apparatus, such as being taken out of the cage, handling, etc. This conclusion may also be supported by a study reporting that behavior subsequent to prior maze experience was not affected by manipulations of extramaze cues (90° reorientation of the maze or use of a different laboratory) [12]. It would be unlikely that the reduction in open arm exploration was due to decreased general locomotor activity because mice with and without test experience did not significantly differ in the distance traveled and number of total arm entries. There was also little possibility that the reduced exploration could be produced by repeated handling by an experimenter during the test battery, as suggested by previous studies indicating that repeated handling increased open arm exploration [51, 52]. A simple explanation of the reduced open arm exploration and increased time spent in the closed arms is that test experience, irrespective of test type, induces an increased level of anxiety. However, given the potential anxiolytic effects of handling [51, 52] in addition to the decreased levels of plasma corticosterone stress response found in this study, it seems to be unlikely that the decreased exploratory behavior reflects increased level of anxiety. Mice with test battery experience showed decreased time spent in the center area, where novelty-seeking and risk-assessment behaviors could be observed [53–55]. These findings suggest another possibility: the decreased open arm exploration in the mice with test battery experience may be due to a reduction in novelty seeking. On the other hand, naïve mice with no prior test experience might exhibit intense anxiety-like behavior similar to panic-like reactions to escape from open arms [54] when confronted with a novel environment for the first time. In this study, the use of the method of picking mice by the tail before the test might also enhance stress and anxiety levels [56, 57], especially in naïve mice, resulting in higher levels of corticosterone stress responses. In our lab, we have analyzed behaviors of more than 200 mutant strains of mice using a battery of behavioral tests. Mice with mutations in genes, such as Calcineurin (CNB, also called protein phosphatase 2B, calcium and calmodulin dependent serine/threonine protein phosphatase), PACAP (pituitary adenylate cyclase-activating polypeptide), PSD-95 (postsynaptic density protein 95), Hivep2 (human immunodeficiency virus type I enhancer binding protein 2, also known as Schnurri-2), Grin1 (glutamate ionotropic receptor NMDA type subunit 1), Reln (reelin), Dab1 (disabled 1), Ts1Cje (reciprocal translocation in chromosome 16), Caskin1 (CASK interacting protein 1), and Syngap1 (synaptic Ras GTPase activating protein 1), showed more open arm exploration behaviors than their wild-type control mice in the elevated plus maze test, while in the light/dark transition test the mutants exhibited decreases in the time spent in the light chamber and in the number of transitions between the light and dark chambers, which are in general considered to reflect increased anxiety, or no behavioral differences in the light/dark transition test compared to control mice [42, 58–66]. Such paradoxical results are also observed in middle-aged wild type mice compared to young mice [67]. These findings highlight the difficulty in interpreting the increased time and entries in open arms simply as decreased levels of anxiety in some cases and indicate the need for further study to explore what the behaviors mean.

Previous studies have indicated that exploration to the open arms was higher when tested in a maze with closed arms with acrylic transparent/translucent walls than with black acrylic/wooden opaque walls in rats [25–27] and mice [28]. The results of this study are consistent with the previous findings [25–28]. The present study further indicate the importance of the transparency and color of walls on elevated plus maze behavior: the most pronounced differences in the behaviors were found between the clear and black wall conditions, whereas such differences were not observed between the transparent blue wall and black wall conditions, and there were slight differences in open arm exploration between the two types of transparent wall arms and between the two types of opaque closed arms. These behavioral differences do not seem to be related to locomotor activity, since there were no significant wall color-induced changes in distance traveled and number of total arm entries. Rodents have a tendency to avoid open spaces by visual perception, allowing them to discriminate open and closed arms [7, 68]. Our results suggest that when less transparent and more dark-colored closed arms, especially black closed arms, are used, mice can more easily discriminate the open and closed arms and potentiate them to avoid the open arms.

It has been reported that animals tested under high illumination conditions show reduced exploratory behavior in open arms or increased anxiety-like behavior in several previous studies comparing different illumination conditions on the maze floor (20 lx vs. 1200 lx [29]) and on the central platform (44 lx vs. 600 lx [23]; 0, 1, 3, 10, 30, 100, and 300 lx [31]), on the arms (80 and 90 lx on open and closed arms vs. 220 and 110 lx on open and closed arms [30]; 4 lx on all arms vs. 210 and 60 lx on open and closed arms [69]), and in the testing room (0.1 footcandle light condition vs. 20.0 footcandle light condition [32]), while some reports have shown no behavioral differences between animals tested under 30, 300, and 900 lx on arms [33] and 9 and 297 lx in the testing room [34]. Such inconsistent findings may be partially explained by differences in illumination levels between the open and closed arms; a study showed that the difference in illumination levels between the open and closed arms is a determinant of open arm exploration [70], and therefore, lack of behavioral differences between the different lighting conditions [33] might be explained as a consequence of the same illumination levels in all the arms in each lighting condition for rats [70]. The present study showed no significant behavioral differences between the groups of mice tested under the three lighting conditions, although the differences in illumination levels between the open and closed arms differed for the 5-, 100-, and 800-lx illumination conditions (see Additional File 6: Table 1) in C57BL/6J mice. These findings suggest the need to further investigate the effects of illumination levels on elevated plus maze behaviors in mice while considering possible methodological differences between studies, such as species, strain, prior experience, and apparatus construction.

The acute effects of corticosterone on anxiety-related behavior remain controversial, since a single corticosterone administration has shown to have an anxiogenic- and anxiolytic-like effects in the elevated plus maze test and the light/dark transition test [71–73]. A correlation study reported that plasma corticosterone levels in response to exposure to the elevated plus maze were correlated with risk-assessment behaviors and time spent in closed arms in Swiss-Webster mice [39]. However, the present study showed that, although a significant correlation between plasma corticosterone levels and time spent in closed arms was found in the cohort of mice with and without prior test experience, our data from the total group of 116 mice revealed no correlations of plasma corticosterone level with behavior. These observations suggest that there are no clear relationships between the two measures in C57BL/6J mice. One possible limitation of this study is the use of the 10-min test protocol. Considering the higher corticosterone levels in rats exposed to the maze for the 10-min period compared with the 5-min period [37], the lack of correlations in this study might be due to a possible ceiling effect by the 10-min exposure to the maze on corticosterone levels.

The present study reveals that previous experience with certain behavioral tests result in alterations in both elevated plus maze behaviors and plasma corticosterone response, and the closed arm type but not illumination level also had significant effects on behaviors in the elevated plus maze, in C57BL/6J mice. These results emphasize the need for careful consideration of test conditions in interpreting the behavioral outcomes under different test conditions of the elevated plus maze. One limitation of this study was that only male mice were used. It will be necessary to consider the use of females in designing experiments to further understand the influences of various variables, including sex and their interactions, on elevated plus maze behavior. Importantly, higher percentages of open arm time and entries are conventionally interpreted as decreased levels of anxiety. As discussed above, there is the possibility that the increased time and entries in open arms may rather reflect a heightened state of anxiety, escape behavior, and panic-like response to a novel environment in some specific cases. These findings suggest the need to measure anxiety-like behaviors under various situations with different types of tests to avoid misinterpretation and generalization from a single test.

## Supplementary Information

**Additional File 1:** Data file

**Additional File 2: Fig. 1.** Apparatus for the elevated plus maze test. (a) Elevated plus maze apparatus and (b) closed arms with different wall colors; clear, transparent blue, white, and black.

**Additional File 3: Fig. 2.** Basal plasma corticosterone levels in male C57BL/6J mice with or without test experience. Male C57BL/6J mice were subjected to a series of behavioral tests, including an assessment of general health and neurological function, a light/dark transition test, and an open field test. Two days after the open field test, blood was collected from mice with test battery experience (n =8) and from naïve mice with no test experience (n = 8). All of the mice were left undisturbed in their home cages before blood collection. Plasma corticosterone concentrations (ng/mL) were measured. Values are the means ± SEM.

**Additional File 4: Fig. 3.** The effects of intertest interval on behaviors in the elevated plus maze in male C57BL/6J mice. In the first cohort of mice, the test-battery-experience group (n = 16) was subjected to the elevated plus maze test 2, 3, or 4 days after the open field test (the test battery was started on test day 1; open field test was on test day 6; elevated plus maze test was on test day 8, n = 4; test day 9, n = 8; test day 10, n = 4). The naïve mice in the no-test-experience group (n = 16) were tested in the same manner as the test-battery-experience group on the test days (test day 8, n = 8; test day 9, n = 4; test day 10, n = 4). Values are the means ± SEM.

**Additional File 5: Fig. 4.** Relationships between elevated plus maze behaviors and plasma corticosterone levels in male C57BL/6J mice. Scatterplots of elevated plus maze behaviors and plasma corticosterone levels in mice with or without test experience (a–f; n = 32 in total), mice tested in the maze with closed arms with different colored walls (g–l; n = 48 in total), mice tested under different illumination levels (m–r; n = 36 in total), and all mice (s–x; n = 116 in total). Correlations (Spearman’s rank correlation coefficient or rs, and p value) of plasma corticosterone levels with (a, g, m, s) distance traveled (cm), (b, h, n, t) number of total arm entries, (c, i, o, u) time spent in closed arms (s), (d, j, p, v) time spent in center (s), (e, k, q, w) percentage of open arm entries (%), and (f, l, r, x) percentage of open arm time (%) were calculated.

**Additional File 6: Table 1.** Illumination levels in the elevated plus maze of closed arms with different color walls and under different light conditions. For the second group of mice, the illumination level was set to 100 lx at the center of the maze with closed arms with transparent blue walls. Measurements of illumination levels at each location of the maze (central platform, end of the open arms, and end of the closed arms) were repeated three times, and the average was calculated. For the third group of mice, the illumination level was set to 5, 100, or 800 lx at the center of the maze with closed arms with transparent blue walls. *The measurement sensor of the illuminometer was placed on the floor of the central platform and the end of the floor of the open arm horizontally. To measure the illumination level in the closed arm, the sensor was tilted due to the size of the illuminometer.

## Data Availability

All the data directly associated with the results of this study are included in the Data file. The data from the behavioral tests are also accessible through the online database “Mouse Phenotype Database” (http://www.mouse-phenotype.org/).
