# Supplementary figures and images for: Effects of test experience, closed-arm wall color, and illumination level on behavior and plasma corticosterone response in an elevated plus maze in male C57BL/6J mice: a challenge against conventional interpretation of the test
Source: Mol Brain. 2021 Feb 15;14:34. doi: 10.1186/s13041-020-00721-2 (PMC7885464; doi:10.1186/s13041-020-00721-2)

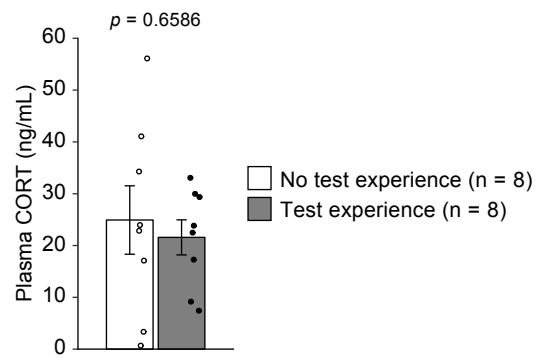

Supplement: Supplementary file 3 — Additional File 3: Fig. 2. Basal plasma corticosterone levels in male C57BL/6J mice with or without test experience. Male C57BL/6J mice were subjected to a series of behavioral tests, including an assessment of general health and neurological function, a light/dark transition test, and an open field test. Two days after the open field test, blood was collected from mice with test battery experience (n =8) and from naïve mice with no test experience (n = 8). All of the mice were left undisturbed in their home cages before blood collection. Plasma corticosterone concentrations (ng/mL) were measured. Values are the means ± SEM. [file 13041_2020_721_MOESM3_ESM.pdf]
